# Multiple QTL Mapping in Autopolyploids: A Random-Effect Model Approach with Application in a Hexaploid Sweetpotato Full-Sib Population

**DOI:** 10.1534/genetics.120.303080

**Published:** 2020-05-05

**Authors:** Guilherme da Silva Pereira, Dorcus C. Gemenet, Marcelo Mollinari, Bode A. Olukolu, Joshua C. Wood, Federico Diaz, Veronica Mosquera, Wolfgang J. Gruneberg, Awais Khan, C. Robin Buell, G. Craig Yencho, Zhao-Bang Zeng

**Affiliations:** * Bioinformatics Research Center, North Carolina State University, Raleigh, North Carolina 27695; † Department of Horticultural Science, North Carolina State University, Raleigh, North Carolina 27695; ‡ International Potato Center, ILRI Campus, Nairobi, Kenya 25171-00603; § Department of Entomology and Plant Pathology, University of Tennessee, Knoxville, Tennessee 37996; ** Department of Plant Biology, Michigan State University, East Lansing, Michigan 48824; †† International Potato Center, Peru, Lima 1558; ‡‡ Plant Pathology and Plant-Microbe Biology Section, Cornell University, Geneva Campus, New York 14456

**Keywords:** multiple interval mapping, polyploid QTL model, restricted maximum likelihood, variance components, yield components, heritability

## Abstract

In developing countries, the sweetpotato, *Ipomoea batatas* (L.) Lam. (2n=6x=90), is an important autopolyploid species, both socially and economically. However, quantitative trait loci (QTL) mapping has remained limited due to its genetic complexity. Current fixed-effect models can fit only a single QTL and are generally hard to interpret. Here, we report the use of a random-effect model approach to map multiple QTL based on score statistics in a sweetpotato biparental population (‘Beauregard’ × ‘Tanzania’) with 315 full-sibs. Phenotypic data were collected for eight yield component traits in six environments in Peru, and jointly adjusted means were obtained using mixed-effect models. An integrated linkage map consisting of 30,684 markers distributed along 15 linkage groups (LGs) was used to obtain the genotype conditional probabilities of putative QTL at every centiMorgan position. Multiple interval mapping was performed using our R package QTLpoly and detected a total of 13 QTL, ranging from none to four QTL per trait, which explained up to 55% of the total variance. Some regions, such as those on LGs 3 and 15, were consistently detected among root number and yield traits, and provided a basis for candidate gene search. In addition, some QTL were found to affect commercial and noncommercial root traits distinctly. Further best linear unbiased predictions were decomposed into additive allele effects and were used to compute multiple QTL-based breeding values for selection. Together with quantitative genotyping and its appropriate usage in linkage analyses, this QTL mapping methodology will facilitate the use of genomic tools in sweetpotato breeding as well as in other autopolyploids.

## Abstract (precis)

Genetic analysis in autopolyploids is a very complicated subject due to the enormous number of genotypes at a locus that needs to be considered. For instance, the number of...

GENETIC analyses in polyploid species pose extra challenges in comparison with diploid species, in spite of the evolutionary benefits that duplication of whole sets of chromosomes may have brought (Comai 2005; Van de Peer *et al.* 2009). When it comes to molecular markers, a codominant, biallelic single nucleotide polymorphism (SNP) directly informs on the genotypes of a diploid locus, but the best it can do alone in a polyploid locus is to inform on its allele dosage. In diploid species, molecular markers are usually scored qualitatively, and there are several methodologies and tools for performing linkage (*e.g.*, Stam 1993; Margarido *et al.* 2007) and quantitative trait loci (QTL) analyses (*e.g.*, Broman *et al.* 2003; Da Costa E Silva *et al.* 2012a). In allopolyploid species, such as cotton (Wu *et al.* 2015) and wheat (Hulse-Kemp *et al.* 2015), where preferential paring dictates meiotic chromosome behavior much like diploids, existing approaches can be readily applied. However, despite many successful studies in diploids and allopolyploids, QTL mapping in autopolyploids remains difficult. In fact, unlike diploid mapping populations, which can have two to four segregating QTL genotypes (in case of inbred or outbred species, respectively), autopolyploid mapping populations can have a much wider range of possible genotypes per locus. For example, there are up to 36, 400, or 4900 possible genotypes from crosses between two tetra-, hexa-, or octoploid outbred parents, respectively.

Single-dose markers, segregating in a 1:1, 3:1, or 1:2:1 fashion, have limited information for building integrated genetic maps in autopolyploids, and generally result in either separate parental maps (*e.g.*, Shirasawa *et al.* 2017) or limited map integration (*e.g.*, Balsalobre *et al.* 2017). In order to make use of multiple-dose markers, the first step is to perform dosage or quantitative SNP calling. Although most methods were designed for tetraploid species (*e.g.*, Voorrips *et al.* 2011; Schmitz Carley *et al.* 2017), additional studies have produced methods that can analyze data for higher ploidy levels (Serang *et al.* 2012; Gerard *et al.* 2018). For building integrated genetic maps in tetraploid species, one can use the well-established TetraploidSNPMap (Hackett *et al.* 2016) as well as TetraOrigin (Zheng *et al.* 2016), which also considers multivalent pairing. For higher ploidy species, MAPpoly (Mollinari and Garcia 2019) is a better option than polymapR (Bourke *et al.* 2018), because the former has implemented a hidden Markov model (HMM) general enough to analyze higher ploidy levels, whereas the latter is limited to tetra- and hexaploid species, and lacks HMM implementation to robustly map all multiple-dose markers (see Mollinari *et al.* 2020). With an integrated map, one can calculate the genotype conditional probabilities of putative QTL, ideally using appropriate HMM (Hackett *et al.* 2016; Mollinari and Garcia 2019). Based on a polyploid model in Kempthorne (1955), a single-QTL model, hereinafter referred to as fixed-effect interval mapping (FEIM), was proposed for autotetraploids (Hackett *et al.* 2001, 2014), and later extended for autohexaploids (van Geest *et al.* 2017).

For an even ploidy level *m*, the FEIM model can be written as

$$y_{i}=\mu \prime +\sum_{j=2}^{m}\alpha_{j}X_{ij}+\sum_{j=m+2}^{2m}\alpha_{j}X_{ij}+\varepsilon_{i}$$ (1)

where *y_i_* is the phenotypic value of individual *i*, *μ*′ is the intercept, *α_j_* is the additive effect of allele *j*, *X_ij_* is the conditional probability of allele *j* in individual *i*, and $\varepsilon_{i}$ is the residual error. The constraints *α*_1_ = 0 and $\alpha_{m+1}=0$ are naturally imposed to satisfy the conditions $\sum_{j=1}^{m}X_{j}=m/2$ and $\sum_{j=m+1}^{2m}X_{j}=m/2$, so that *μ*′ is a constant that is hard to interpret due to these constraints (Hackett *et al.* 2001, 2014). Note that 2*m*−2 additive allele effects need to be estimated, *i.e.*, tetra-, hexa-, or octoploid models will have 6, 10, or 14 main effects, respectively. In order to test whether the additive allele effects are different from zero (the null hypothesis), likelihood-ratio tests (LRT) are performed along positions on a genetic map. Commonly, the tests are presented as “logarithm of the odds” (LOD scores), where LOD=LRT/[2×ln(10)]. In order to declare a QTL, empirical LOD thresholds are computed for each trait using permutations (Churchill and Doerge 1994). This approach has been used widely so far (*e.g.*, Schumann *et al.* 2017; van Geest *et al.* 2017; Massa *et al.* 2018). However, limitations in fitting multiple-QTL models have been presented, due mostly to the possibility of over-parameterization or the lack of optimized algorithms for model selection (Mengist *et al.* 2018; Klaassen *et al.* 2019).

Variance component methods have been used for performing QTL mapping in related individuals of complex population structures or families in humans (Lippert *et al.* 2014), animals (Druet *et al.* 2008), and plants (Crepieux *et al.* 2005). In common, these approaches take into account the flexibility of mixed models in dealing with the correlated QTL genotype effects among individuals due to shared alleles identical-by-descent (IBD) by each relative pair at a particular location in the genome. Since a higher ploidy level leads to a much larger number of allele combinations, genotypic effects may be very hard to assess from the small population sizes usually available. In this case, the integrated genetic map provides key information on the inheritance of sets of chromosomal segments from parents to progeny (Mollinari and Garcia 2019), making up the basis for IBD-based additive relationship estimation. If a locus is linked to a region underlying the variation of a trait of interest, more shared alleles IBD for that locus are expected among individuals with similar phenotypic values (Almasy and Blangero 2010). Thus, the key parameters in this model are the variance components attributable to putative QTL, which determine the presence of linkage. Because only one parameter per QTL (the variance component) needs to be estimated, one could try to build a multiple-QTL model for polyploids, inspired by the corresponding multiple interval mapping (MIM) for diploid mapping populations (Kao *et al.* 1999), without the risk of model over-parameterization.

A multiple-QTL mapping approach is expected to increase detection power, enable separation of linked QTL, and provide the basis for studying QTL interaction (epistasis). Thus, such a model may benefit several autopolyploid horticultural (*e.g.*, potato, blueberry, kiwifruit, strawberry), ornamental (*e.g.*, rose, chrysanthemum), forage (*e.g.*, alfalfa, guinea grass), and field (*e.g.*, sugarcane) crops. The sweetpotato [*Ipomoea batatas* (L.) Lam. (2n=6x=90)] is a staple food in several developing countries, with a production of 112 million tons worldwide in 2017 (FAO 2019). Particularly, it has attracted growing interest due to its characteristics for food and nutrition security (Mwanga *et al.* 2017). In addition to carbohydrates, dietary fiber, vitamins, and minerals, orange-fleshed sweetpotatoes provide high levels of *β*-carotene to fight vitamin A deficiency in vulnerable populations, such as those in sub-Saharan Africa (Low *et al.* 2017). In order to increase production and meet farmer’s and market needs, it is imperative to make molecular-assisted selection an effective part of sweetpotato breeding programs. Toward this end, one of the first steps is to characterize the genetic architecture of traits of interest, such as those related to storage root yield and quality, and resistance to biotic and abiotic stresses (Khan *et al.* 2016). In spite of being considered an “orphan” crop, there have been recent advances in building genome references from its wild diploid relatives (Wu *et al.* 2018), optimizing a genotyping-by-sequencing protocol (GBSpoly) for high-throughput SNP genotyping (Wadl *et al.* 2018), and building a high-density integrated genetic map (Mollinari *et al.* 2020). In this paper, we introduce a random-effect multiple interval mapping (REMIM) model for autopolyploids. Using a genome-assisted, GBSpoly-based integrated genetic map from a sweetpotato biparental population, we map QTL for yield-related traits with our open-source software, QTLpoly.

## Materials and Methods

### Full-sib population

A bi-parental mapping population (named BT) comprising 315 individuals was developed by crossing an orange-fleshed American variety, ‘Beauregard’ (CIP440132), and a nonorange-fleshed African landrace, ‘Tanzania’ (CIP440166), as male and female parents, respectively. The parents show contrasting phenotypes for several traits such as dry matter, *β*-carotene and sugar content, and susceptibility to biotic (*e.g.*, virus disease) and abiotic (*e.g.*, drought) stresses. ‘Beauregard’ is known as to have higher yield than ‘Tanzania’, and the current QTL mapping study will focus on yield components.

### Phenotypic analyses

#### Field trials:

In addition to the 315 full-sibs, parents (each replicated twice) and another variety, ‘Daga’ (CIP199062.1), were used as checks, making up a total of 320 individuals per replication in an 80 × 4 alpha-lattice design. Virus-free planting material derived from tissue culture was obtained from the CIP-Peru Genebank in La Molina. The clones were grown in a screen house in CIP substation San Ramon, and the planting material multiplied under low-disease pressure field conditions in Satipo, where cuttings for the six experiments were obtained. Four experiments were conducted in Ica (14°01′ S and 75°44′ W, 420 m), with two independent trials over two seasons, and one experiment each was conducted in San Ramon (11°07′ S and 75°21′ W, 828 m) and Pucallpa (8°23′ S and 74°31′ W, 154 m). The number of replications were two at Ica and three at San Ramon and Pucallpa. In all trials, 1 m and 0.3 m of inter- and intra-row spacing was used, respectively. In the first season at Ica (from 25 February to 29 June 2016), the plot size was 6 m^2^ of 16 plants arranged in four rows (4 plants per row) with one empty row between plots. In the second season at Ica (from 15 November 2016 to 17 March 2017), the plot size was 4.8 m^2^ of 16 plants arranged in two rows (8 plants per row) with no empty row between plots. In San Ramon (from 14 May to 15 September 2016) and Pucallpa (from 1 July to 4 November 2016), the plot size was 9 m^2^ of 30 plants arranged in three rows (10 plants per row) with no empty row between plots.

#### Phenotypic data:

Eight yield-related phenotypes (see File S1) were collected per plot at harvest, ∼120 days after transplanting. For analysis purposes, foliage and root yield data were standardized by plot size (relative to the largest) and converted to tons per hectare (t ha^−1^) to allow comparisons across trials. Number of roots was divided by the number of plants in the plot. The total number of storage roots per plant (TNR) and total root yield (RYTHA) considered all storage roots from the whole plot regardless of their individual weight. Number of commercial roots per plant (NOCR) and commercial root yield (CYTHA) considered only storage roots of marketable size (≥100 g for African market). Number of noncommercial roots per plant (NONC) and noncommercial root weight (NCYTHA) were obtained from the difference between total and commercial roots. Foliage yield (FYTHA) was measured by weighing all above-ground biomass per plot. Finally, commercial index (CI) was calculated as the ratio between CYTHA and total biomass (*i.e.*, the sum of RYTHA and FYTHA).

#### Multi-environment phenotypic model:

We considered each one of the six field trials as an environment. Jointly adjusted means for each individual were obtained by using the following mixed-effect model

$$y_{ijkl}=\mu +e_{l}+r_{k\left(l\right)}+b_{j\left(kl\right)}+g_{i}+ge_{il}+\varepsilon_{ijkl}$$ (2)

where *y_ijkl_* is the phenotypic observation of the *i*^th^ genotype in the *j*^th^ block within the *k*^th^ replicate at the *l*^th^ environment, *μ* is the overall mean, *e_l_* is the random effect of the *l*^th^ environment (l=1,…,L; L=6) with $e_{l}∼N\left(0,\sigma_{e}^{2}\right)$, $r_{k\left(l\right)}$ is the random effect of the *k*^th^ replicate (k=1,…,K; K=2 or 3 depending on the environment) at the *l*^th^ environment with $r_{k\left(l\right)}∼N\left(0,\sigma_{r}^{2}\right)$, $b_{j\left(kl\right)}$ is the random effect of the *j*^th^ block (j=1,…,J; J=80) within the *k*^th^ replicate at the *l*^th^ environment with $b_{j\left(kl\right)}∼N\left(0,\sigma_{b}^{2}\right)$, *g_i_* is the fixed effect of the *i*^th^ genotype (i=1,…,I; I=318), *ge_il_* is the random effect of genotype-by-environment (G × E) interaction with $ge_{il}∼N\left(0,\sigma_{ge}^{2}\right)$, and *ε_ijkl_* is the random residual error with $\varepsilon_{ijkl}∼N\left(0,\sigma_{l}^{2}\right)$ (*i.e.*, environment specific variances). Variance components were estimated by restricted maximum likelihood (REML) using ASReml-R (v4.1; https://www.vsni.co.uk/software/asreml-r).

Mean-basis broad-sense heritabilities (*H*^2^) were approximate as the ratio between genotypic and phenotypic variances as

$$H^{2}=\frac{\sigma_{g}^{2}}{\sigma_{g}^{2}+\frac{\sigma_{ge}^{2}}{L}+\frac{\sigma_{\varepsilon }^{2}}{\bar{K}L}}$$

where $\sigma_{g}^{2}$ is the variance component associated with the *g*_i_ term from Equation 2 when treated as a random effect, *i.e.*, $g_{i}∼N\left(0,\sigma_{g}^{2}\right)$, $\sigma_{\varepsilon }^{2}$ is the variance component associated with the residual error but with a common variance for all environments, *i.e.*, $\varepsilon_{ijkl}∼N\left(0,\sigma_{\varepsilon }^{2}\right)$, and $\bar{K}=2.25$ is the harmonic mean of the number of replicates across environments.

Finally, Pearson’s correlations (significance *P < 0.05, **P < 0.01 and ***P < 0.001) were computed among the individual adjusted means.

### Genotypic analyses

#### GBSpoly and dosage calling:

A modified GBS protocol called GBSpoly was carried out according to Wadl *et al.* (2018) and described in detail for the BT population by Mollinari *et al.* (2020). In brief, total DNA was extracted and double restricted using a *Cvi*AII-*Tse*I enzyme combination for all full-sibs and parents (each parent replicated 10 times). Restriction fragments were ligated to adapters, size selected, and amplified. Adapters contained an 8-bp buffer sequence in addition to sample-specific variable length barcodes (6–9 bp). Each 64-plex library was sequenced using eight lanes of an Illumina HiSeq 2500 system in order to ensure optimal read depth for dosage calling. We trimmed the 8-bp buffer sequence from the reads using the FASTX-Toolkit (available at hannonlab.cshl.edu/fastx_toolkit/). A modified version of Tassel-GBS pipeline (v4.3.8), called Tassel4-Poly (Pereira *et al.* 2018, available at https://github.com/gramarga/tassel4-poly) was used to demultiplex and to count and store the actual read depth for all loci in variant call format (VCF) files. We used Bowtie2 (Langmead and Salzberg 2012) to align 64-bp tags against the *I. trifida* and *I. triloba* genomes, two sweetpotato wild relative diploid species (Wu *et al.* 2018, available at http://sweetpotato.plantbiology.msu.edu). Finally, the software SuperMASSA (Serang *et al.* 2012, available at https://bitbucket.org/orserang/supermassa) was used to perform multi-threading dosage call through a wrapper function named VCF2SM (Pereira *et al.* 2018, available at https://github.com/gramarga/vcf2sm).

#### Linkage mapping:

A linkage map was constructed by Mollinari *et al.* (2020) using the R package MAPpoly (Mollinari and Garcia 2019, available at https://github.com/mmollina/mappoly; see File S2). In brief, we computed two-point recombination fractions between all 38,701 nonredundant, high quality GBSpoly-based markers, and sorted the most likely linkage phase between each marker pair. Markers were then grouped into 15 linkage groups (LGs) by using the Unweighted Pair Group Method with Arithmetic Mean (UPGMA) hierarchical clustering method. For each LG, large-scale ordering was obtained using multidimensional scaling as implemented in the R package MDSMap (Preedy and Hackett 2016), and then small-scale ordering was refined based on the reference genomes (see details in Mollinari *et al.* 2020). Map distances, computed using Haldane’s map function, were re-estimated based on the individual posterior probabilities from SuperMASSA dosage calls. The final integrated, completely phased map was composed of 30,684 markers distributed along 15 LGs with a total length of 2708.3 centiMorgans (cM) and no major gaps between markers (11.3 markers every cM, on average). Multi-point genotype conditional probabilities of putative QTL were estimated for every individual given the final map using an HMM algorithm (Lander and Green 1987; Jiang and Zeng 1997) adapted for polyploids (Mollinari *et al.* 2020) as implemented in MAPpoly. Since 17 full-sibs were filtered out along the map construction (Mollinari *et al.* 2020), only the remaining 298 individuals were ultimately used for QTL mapping.

### QTL mapping analyses

Under random bivalent pairing, an autopolyploid individual of a species with an even ploidy level *m* can produce $\left(\begin{array}{c} m\\ m/2 \end{array}\right)$, or “*m* choose m/2”, different gametes with the same probability, and a cross between two individuals can generate $p={\left(\begin{array}{c} m\\ m/2 \end{array}\right)}^{2}$ different genotypes. As an example, consider two contrasting parents, A and B, of a hexaploid species (such as sweetpotato) and their respective genotypes for a QTL as *abcdef* and *ghijkl*, each one with potentially six different alleles. As each parent can produce 20 different gametes, the cross A × B would generate $p=20^{2}=400$ possible different genotypes. The model detailed next can be adapted easily to any polyploid species with an even ploidy level by simply changing *p* accordingly, *e.g.*, $p=6^{2}=36$ for autotetraploid and $p=70^{2}=$ 4,900 for autooctoploid full-sib families.

#### REMIM model and hypothesis testing:

Taking a full-sib population with *n* individuals derived from a cross between two hexaploid parents, A and B, the multiple-QTL mapping model is expressed by

$$y=1\mu +\sum_{q=1}^{Q}Z_{q}u_{q}+\varepsilon$$ (3)

where ***y*** is the *n*×1 vector of phenotypic values (in our case, the jointly adjusted means from the phenotypic analysis), *μ* is the fixed effect of population mean, ***u****_q_* is the *p*×1 random vector of additive genetic values of QTL *q* (q=1,…,Q) with $u_{q}∼N\left(0,\Pi \sigma_{q}^{2}\right)$, and ***ε*** is the *n*×1 random vector of residual error with $\varepsilon ∼N\left(0,I\sigma^{2}\right)$. **1** and I are an *n*×1 vector of 1’s and an *n*×*n* identity matrix, respectively, ***Z****_q_* is the *n*×*p* incidence matrix of genotype conditional probabilities of QTL *q*, and **Π** is a *p*×*p* additive relationship matrix between the *p* possible QTL genotypes. This matrix is fixed for a given ploidy level, and, for a hexaploid species, **Π** cells assume one out of seven different values ($\Pi_{jj\prime }=\left\{0/6,1/6,2/6,3/6,4/6,5/6,6/6\right\}$ with j,j′={1,…,p}), depending on how many alleles IBD are shared between two genotypes. For example, the genotype pair *abcghi-defjkl* shares 0 out of 6 alleles, hence $\Pi_{jj\prime }=0/6$, whereas *abcghi-abcghi* shares 6 out of 6 alleles, hence $\Pi_{jj\prime }=6/6$.

Assuming that the random-effect QTL are uncorrelated, each with expectation zero, the expectation of the vector of phenotypic values ***y*** is

E(y)=1μ

and its variance-covariance matrix is

$$\text{Var}\left(y\right)=V=\sum_{q=1}^{Q}G_{q}\sigma_{q}^{2}+I\sigma^{2}$$

where $G_{q}=Z_{q}\Pi Z_{q}\prime$ is the n×n additive relationship matrix between the *n* individuals on the putative QTL *q*, and $\sigma_{q}^{2}$ and $\sigma^{2}$ are the respective variance components associated with QTL *q* and the residual error. In other words, each $G_{q}$ cell $G_{ii\prime \left(q\right)}$ ranges from 0 to 1, as the result of the sum over the products of QTL genotype probabilities, $Z_{ij\left(q\right)}=\text{Pr}\left(G_{ij\left(q\right)}|\text{map}\right)$ and $Z_{i\prime j\prime \left(q\right)}=\text{Pr}\left(G_{i\prime j\prime \left(q\right)}|\text{map}\right)$, from ***Z****_q_* of a sib-pair *i* and *i*^′^ (i,i′={1,…,n}), weighted by the proportion of shared alleles IBD $\Pi_{jj\prime }$ from **Π** between each pair of possible genotypes *j* and *j*′ (j,j′={1,…,p}), *i.e.*

$$\begin{array}{l} G_{ii^{\prime }\left(q\right)}=\sum_{j,j\prime =1}^{p}\text{Pr}\left(G_{ij\left(q\right)}|\text{map}\right)\text{Pr}\left(G_{i\prime j\prime \left(q\right)}|\text{map}\right)\Pi_{jj\prime }\\ =\sum_{j,j\prime =1}^{p}Z_{ij\left(q\right)}Z_{i\prime j\prime \left(q\right)}\Pi_{jj\prime } \end{array}$$

If the respective genotypes *j* and *j*′ of two sibs *i* and *i*′, $G_{ij\left(q\right)}$ and ${\mathrm{script}}_{i\prime j\prime \left(q\right)}$, are observed with certainty, computing pairwise additive relationship is straightforward, *e.g.*, $\text{Pr}\left(G_{i1\left(q\right)}=abcghi|\text{map}\right)=1$ and $\text{Pr}\left(G_{i\prime 2\left(q\right)}=abcghj|\text{map}\right)=1$, hence $G_{ii\prime \left(q\right)}=5/6$. However, if the probability of the genotype of *i* is split in two, *e.g.*, $\text{Pr}(G_{i1\left(q\right)}=abcghi|\text{map})=1/2$ and $\text{Pr}(G_{i2\left(q\right)}=abcghj|\text{map})=1/2$, then $G_{ii\prime \left(q\right)}=1/2\cdot 1\cdot 5/6+1/2\cdot 1\cdot 6/6=5.5/6$. It is interesting to note that, if ***G****_q_* is averaged out for all map positions, $G_{ii\prime }\approx 1\;\forall \;i=i\prime$ (an individual with itself), and $G_{ii\prime }\approx 1/2\;\forall \;i\neq i\prime$ (any sib pair), consisting of the realized additive relationship matrix.

Here, our interest is in testing

$$H_{0}:\sigma_{q}^{2}=0\text{vs}.\;H_{a}:\sigma_{q}^{2}>0$$

*i.e.*, whether QTL *q* contributes to the variation in ***y*** or not, so that several tests have to be performed along the genome. As part of the algorithm described next, we test for the presence of multiple QTL in consecutive rounds. In practice, we compute and store a ***G****_q_* matrix for every putative QTL *q*, representing genomic positions at a certain step size (*e.g.*, every 1 cM). In this case, Equation 3 can be rewritten as

$$y=1\mu +\sum_{q=1}^{Q}g_{q}+\varepsilon$$ (4)

where ***g****_q_* is an *n*×1 random vector of the individual breeding values on the QTL *q* with $g_{q}∼N\left(0,G_{q}\sigma_{q}^{2}\right)$. Notice how Equation 3 and 4 are closely connected. At the QTL peak *q*, ***u****_q_* (Equation 3) are the best linear unbiased prediction (BLUP) values of the *p* QTL genotype effects, whereas ***g****_q_* (Equation 4) are the BLUP values for the *n* full-sibs, *i.e.*, the individual breeding values based on the additive allele effects. Therefore, the QTL-based breeding values ${\hat{g}}_{q}$ are equivalent to those from $Z_{q}{\hat{u}}_{q}$. These alternatives forms are conveniently used in different contexts of QTL detection and characterization, as described next. While Equation 4 is preferred in order to make the multiple-QTL model selection less computational intensive because the stored ***G****_q_* can be used recursively, Equation 3 is used to describe the QTL genotype effects, which are ultimately used to compute additive allele effects.

We computed linear score statistics according to Qu *et al.* (2013) at every position and compared its *P*-value with a prescribed critical value, as part of the algorithm used to declare QTL as described in the next section. In order to compute the test statistics, we assume that ***y*** has normal distribution, *i.e.*, $y∼N\left(1\mu ,V\left(\tau \right)\sigma^{2}\right)$, with the variance components of QTL *q* rescaled in relation to $\sigma^{2}$ as $\sigma_{q}^{2}=\sigma^{2}\tau_{q}$, so that

$$V\left(\tau \right)=G_{r}\tau_{r}+\sum_{q\neq r}G_{q}\tau_{q}+I$$

where *r* is the putative QTL being tested in a model with q=1,…,r,…,Q QTL and $\tau =\left(\tau_{r},\tau_{\left(Q-1\right)}\right)\prime$. Using this form, the hypothesis testing becomes $H_{0}:\tau_{r}=0\text{vs}.\;H_{a}:\tau_{r}>0$, and $\tau_{\left(Q-1\right)}$ components are regarded as nuisance parameters. The other nuisance parameters *μ* and $\sigma^{2}$ are removed when writing the log REML profiled function

$$\ell \left(\tau \right)\propto -\frac{1}{2}\left\{\tilde{n}\text{log}\left[\tilde{y}\prime \tilde{V}{\left(\tau \right)}^{-1}\tilde{y}]+\text{log}[\left|\tilde{V}\left(\tau \right)\right|\right]\right\}$$

from the restricted parameterization of $\tilde{y}=\mathrm{Ay}$, in which ***A*** is an $\tilde{n}\times n$ matrix $\left(\tilde{n}=n-1\right)$, such that ***A*1** = **0** and $\mathrm{AA}\prime =I_{\tilde{n}}$ (Qu *et al.* 2013). Accordingly, it follows that $\tilde{y}∼N\left(0,\tilde{V}\left(\tau \right)\sigma^{2}\right)$, with $\tilde{V}\left(\tau \right)={\tilde{G}}_{r}\tau_{r}+\sum_{q\neq r}{\tilde{G}}_{q}\tau_{q}+I_{\tilde{n}}$ and ${\tilde{G}}_{q}=AG_{q}A\prime$. Based on ℓ(τ), the score function of $\tau_{r}$ is given by $S_{r}\left(\tau \right)=\partial \ell /\partial \tau_{r}$, *i.e.*

$$S_{r}\left(\tau \right)\equiv \frac{\tilde{n}}{2}\frac{\tilde{y}\prime \tilde{V}{\left(\tau \right)}^{-1}{\tilde{G}}_{r}\tilde{V}{\left(\tau \right)}^{-1}\tilde{y}}{\tilde{y}\prime \tilde{V}{\left(\tau \right)}^{-1}\tilde{y}}-\frac{1}{2}\text{tr}\left[\tilde{V}{\left(\tau \right)}^{-1}{\tilde{G}}_{r}\right]$$

(Qu *et al.* 2013).

On the one hand, when there is only one QTL (r=Q=1), *i.e.*, ${\hat{\tau }}_{0}={\hat{\tau }}_{r}=0$ under *H*_0_, *S_r_* (0) is the exact (nonasymptotic) test statistic. On the other hand, a moment-based approximation to the null distribution is used when two or more QTL are present in the model (*Q* > 1), *i.e.*, ${\hat{\tau }}_{0}=\left(0,{\hat{\tau }}_{\left(Q-1\right)}\right)\prime$ under *H*_0_ (Qu *et al.* 2013). The validity of the moment-based approximation was assessed through simulations, as suggested by Qu *et al.* (2013). In any case, large score value indicates departure from *H*_0_. The *P*-values associated with the linear score test are continuous over the unit interval as a result of weighted sums of the scores from the profiled likelihood. Herein, we conveniently take the “logarithm of *P*” as $LOP=-{\text{log}}_{10}\left(P\right)$ for graphic representation and supporting interval calculation purposes. Support intervals are defined as the QTL peak neighboring region with *LOP* greater than, or equal to, *LOP*−*d*, where *d* is a constant that subtracts the highest *LOP* (thus from the QTL peak) in that region, as similarly proposed for the LOD score statistics (Lander and Green 1987).

#### QTL detection and characterization:

In order to select QTL, we adapted the MIM methodology described by Kao *et al.* (1999) to a random-effect model framework as follows:

1. *Forward search* adds one QTL at a time to the model at the position with the highest score statistic if the *P*-value is smaller than a genome-wide significance threshold level (*e.g.*, *α*=0.20), and fits it into the model. Consecutive rounds of search for a new QTL are carried out conditioning to the one(s) in the model until no more QTL positions can reach the threshold. A window size (*e.g.*, of 20 cM) is avoided on either side of QTL already in the model when searching for a new QTL;
2. *Model optimization* follows rounds of position refinement and backward elimination when no more QTL can be added in the forward search step. In turn, a QTL position is updated conditional to all the other QTL in the model, and its score statistic is re-evaluated at a more stringent significance threshold level (*e.g.*, *α*=0.05), when the QTL may be dropped. The final set of QTL is defined when all selected positions are significant, and, thus, no more positions change or QTL are dropped;
3. Forward search (now with a threshold value as stringent as the one used for backward elimination, *e.g.*, *α*=0.05) as well as model optimization procedures are repeated until no more QTL are added (via forward search) or dropped (via backward elimination). Finally, *QTL profiling* is performed with the remaining significant QTL after the last round of model optimization has been carried out. The score statistics and their associated *P*-values are computed for all genomic positions conditional to the final set of QTL.

Notice that, as part of the strategy for selecting QTL, we were less stringent during the first step of *forward search*, so that we were able to allow more positions to be tested again during *model optimization*. In fact, power for detecting significant positions is expected to increase when conditioning the forward search as well as the backward elimination to other QTL already in the model (Da Costa E Silva *et al.* 2012a). For the forward search performed after the first backward elimination, we used the last threshold set from the backward elimination in order to avoid false positives. Here, we adopted the score-based resampling method to assess the genome-wide significance level proposed by Zou *et al.* (2004). In brief, *n* independent samples from a standard normal distribution were used to obtain the score statistic for every map position under evaluation (*e.g.*, every 1 cM) and the *P*-value of the highest score was stored. After repeating this *N* = 1000 times (resampling), the score-based threshold for a significance *α* level was then defined from the 100(1−*α*) percentile of ascending ordered *P*-values from the *N* samples.

Once the QTL were selected, we were able to estimate their variance components and compute QTL heritabilities, $h_{q}^{2}$, as the ratio between the QTL variance component and total variance. Given the parameter estimates, QTL-based breeding values are directly obtained as the BLUPs of the QTL genotypes (*i.e.*, the vector ${\hat{u}}_{q}$) from Equation 3. BLUPs of the *p* = 400 possible genotypes were further decomposed in order to compute the additive effects as the average of ${\hat{u}}_{q}$ (BLUPs) containing the respective alleles. Note that, in an F_1_ population, the allele substitution effects (the very definition of additive effects) can be assessed only among alleles within each parent. Due to the model assumptions of zero mean for random effects, additive allele effects sum up to zero. These effects should be interpreted as the heritable contributions from parent to offspring, hence providing straightforward estimation of QTL-based breeding values to be used for selection.

### Simulations

We conducted the following simulation study to examine the performance of REMIM (Equation 4) and compare it with FEIM (Equation 1). We simulated quantitative traits with three QTL each (q=1,…,Q; Q=3) positioned along the BT linkage map (Mollinari *et al.* 2020, *n* = 298; see File S2) according to three scenarios (1000 simulations each): (*i*) unlinked, where all QTL were positioned in different LGs each; (*ii*) random, where each QTL was positioned randomly, but no closer than 20 cM from each other in case of being assigned to the same LG; and (*iii*) linked, where at least two QTL were positioned in the same LG, but no closer than 50 cM from each other. The QTL heritabilities were simulated as $h_{q}^{2}=\left\{0.3,0.2,0.1\right\}$ following their respective QTL genotype effect distributions as $g_{q}∼N\left(0,G_{q}\sigma_{q}^{2}\right)$, where $\sigma_{q}^{2}=\left\{0.75,0.50,0.25\right\}$. The environmental error was simulated from a standard normal distribution $\left(\sigma^{2}=1\right)$, while the population mean was simulated as zero (μ=0).

One round of *forward search* followed by *model optimization* (steps 1 and 2 from the algorithm described above) was carried out for each simulated trait using different genome-wide significance forward (α=0.20) and backward (α={0.15,0.10,0.05,0.01}) *P*-value thresholds based on the score-based resampling method (Zou *et al.* 2004). For comparison, we ran FEIM (Equation 1) with the same simulated traits using different genome-wide significance LOD thresholds (α={0.20,0.15,0.10,0.05,0.01}) based on 1000 permutations (Churchill and Doerge 1994). We also stored the error vectors used to add noise to each simulated phenotype, and ran FEIM and REMIM models again, now using the respective error vectors as offset variables, which simply subtract the error from its respective phenotype. In this case, we expected that both FEIM and REMIM models would perform similarly, since all the noise had been controlled, so that the only variation left was thus due to the QTL. We used the same step size of 2 cM as well as the same window size of 20 cM for both models. *LOP*−*d* (from REMIM) and *LOD*−*d* (from FEIM) support intervals were calculated for three different *d* values (d={1.0,1.5,2.0}).

Following the definitions and summary statistics from Da Costa E Silva *et al.* (2012b), all QTL kept after model optimization were considered “mapped.” A mapped QTL was considered “paired” if <20 cM apart from the simulated position, and a paired QTL was considered “matched” (true QTL) if its support interval included a simulated QTL. Finally, a mapped QTL was considered “mismatched” (false QTL) if it was not matched. We summarized detection power and empirical false discovery rate (FDR) for each support interval. Power was calculated as the ratio between the number of matched QTL over the total number of simulated QTL. FDR was estimated as the ratio between the number of mismatched QTL over the total number of mapped QTL. The absolute distance differences between simulated and mapped positions of paired QTL (precision) were averaged out. The proportion of matched QTL over the total number of paired QTL as an approximation of support intervals (coverage) was provided for each *d* value.

### Software implementation

We implemented the algorithm for detection and characterization of multiple QTL based on the REMIM model in an R package called QTLpoly (available at https://github.com/guilherme-pereira/qtlpoly). We integrated functions from the R package varComp (v0.2-0; Qu *et al.* 2013) to compute the score statistics. The rounds of QTL search and model optimization use the variance components estimated in the previous round, so that the new estimates iterate faster. In addition, calculations for different genomic positions were paralleled in order to speed up the process by using the R base package parallel (v3.5.2; R Core Team 2019). Final models were fitted using the R package sommer (v3.6; Covarrubias-Pazaran 2016), from which BLUPs were extracted and used for the computation of additive allele effects and QTL-based breeding values. Both varComp and sommer packages use REML estimation to compute the variance components from the random-effect QTL model. Functions for plotting QTL profiles, effects and support intervals were based on ggplot2 (v3.1.0; Wickham 2016). Additional functions for running FEIM model and multi-threaded permutations were included in QTLpoly and were based on the lm() function from R base package stats (v3.5.2; R Core Team 2019).

### Gene expression profiling

A developmental time-course gene expression profiling data of ‘Beauregard’ (NCBI BioProject PRJNA491292) was reported previously (Wu *et al.* 2018), and a parallel time-series of development with ‘Tanzania’ (NCBI BioProject PRJNA549660) roots was recently analyzed, including identification of differentially expressed genes (Gemenet *et al.* 2020). In brief, ‘Beauregard’ and ‘Tanzania’ roots were harvested from four biological replicates at 10, 20, 30, 40, and 50 days after transplanting (DAT), and 30, 40, and 50 DAT roots were classified into fibrous and storage roots based on diameter as described by Wu *et al.* (2018). RNA-sequencing (RNA-seq) datasets from both genotypes were generated and processed as described previously (Lau *et al.* 2018), with the one exception that the ‘Tanzania’ 30 DAT storage root sample was subsampled for 30 million reads. Differentially expressed genes were determined as described in Gemenet *et al.* (2020) using DESeq2 (v1.22.2; Love *et al.* 2014) with a log2 fold-change (*lfc*) threshold of 2 and an adjusted *P*-value cutoff of 0.01, based on the fragments per kilobase exon model per million mapped reads (FPKM). To provide a comparison of expression abundances in the roots to leaves, ‘Beauregard’ and ‘Tanzania’ plants were grown as described in Lau *et al.* (2018) for control conditions and RNA-seq libraries from leaves processed as described above.

### Data availability

The authors state that all data necessary for confirming the conclusions presented in the article are represented fully within the article. Raw RNA-seq reads are available at NCBI under BioProject numbers PRJNA491292 and PRJNA549660. Raw GBSpoly reads and VCF files are available via Mollinari *et al.* (2020). File S1 contains phenotypic data. File S2 contains genetic map information used in this study (also available via Mollinari *et al.* 2020). File S3 is the raw expression abundance matrix (also available via Gemenet *et al.* 2020). File S4 is the log2 FPKM expression matrix (also available via Gemenet *et al.* 2020). File S5 contains the differentially expressed genes associated with this study. MAPpoly software used for linkage mapping analyses is available at GitHub (https://github.com/mmollina/mappoly). QTLpoly software used for QTL mapping analyses and simulations is available at GitHub (https://github.com/guilherme-pereira/qtlpoly). Supplemental material available at figshare: https://doi.org/10.25386/genetics.12246134.

## Results

### Trait heritabilities and correlations

**Figure 1:**
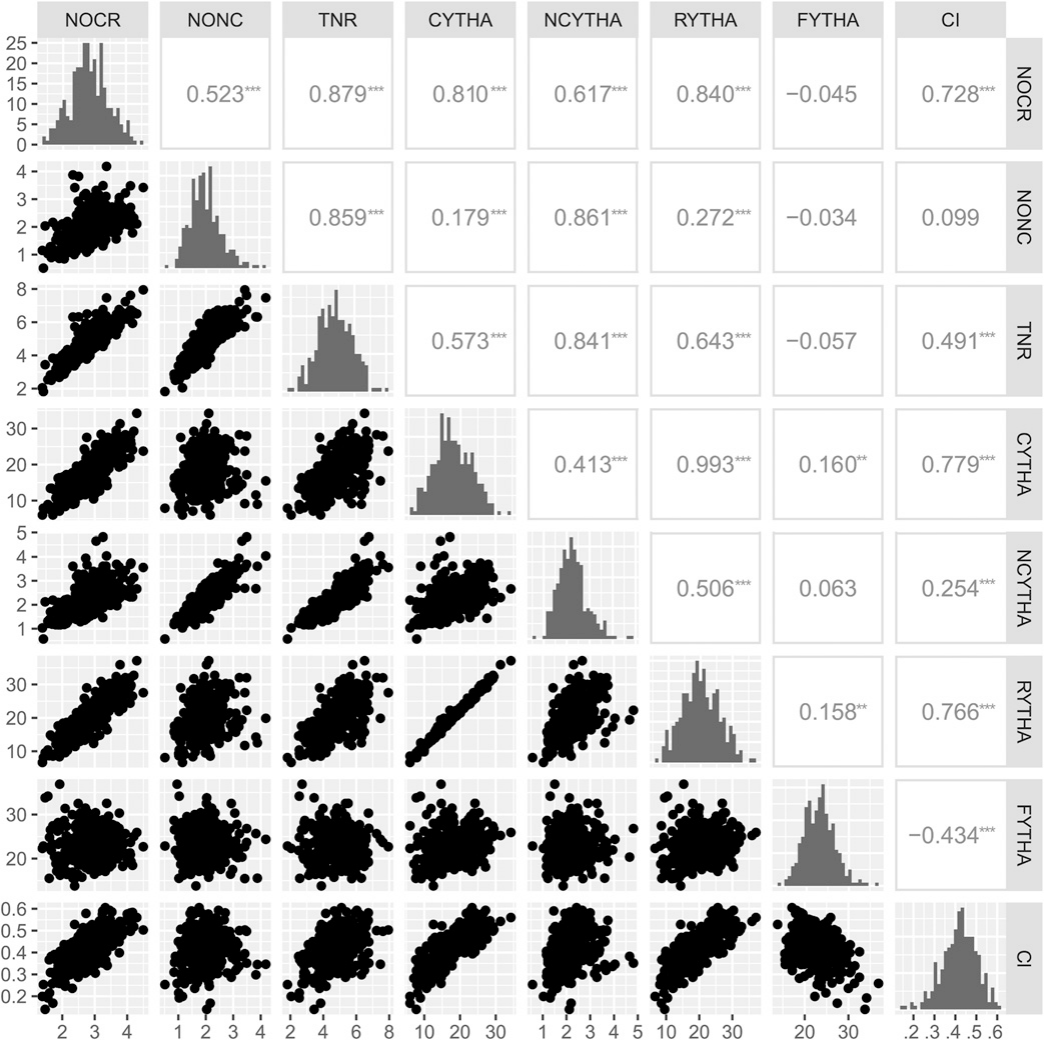
Pearson’s correlations (**P < 0.01, ***P < 0.001) among predicted means of eight yield-related traits from ‘Beauregard’ × ‘Tanzania’ (BT) full-sib family. Trait abbreviations: number of commercial (NOCR), noncommercial (NONC) and total (TNR) roots per plant, commercial (CYTHA), noncommercial (NCYTHA), and total (RYTHA) root yield in t ha^−1^, foliage yield (FYTHA) in t ha^−1^, and commercial index (CI).

Each one of the eight yield-related traits from six environments were analyzed using a multi-environment mixed-effect model, from which we were able to obtain jointly predicted means for each full-sib and variance component estimates (Table 1). Parents showed contrasting means for all traits, with ‘Beauregard’ presenting higher means for number of roots and root yield (both commercial and noncommercial) and commercial index when compared to ‘Tanzania’, which surpassed ‘Beauregard’ only for foliage yield. Interestingly, transgressive segregation was observed among the full-sibs for all traits, with emphasis on several individuals, with CYTHA higher than the most productive parent. Broad-sense heritabilities (*H*^2^) were generally high, ranging from 70.39% (NCYTHA) to 88.42% (CI). Correlations between the predicted means were also estimated (Figure 1). Low to nonsignificant correlations (from −0.04 to 0.16**) were observed between FYTHA and root yield traits. The highest correlation (0.99***) was between CYTHA and RYTHA, which was expected, since most RYTHA is derived from CYTHA. Among the traits used for CI calculation, CYTHA also had the highest correlation with CI (0.78***), likely because it is its main component. TNR components were also highly correlated with TNR, namely NOCR (0.89***) and NONC (0.86***). Finally, NOCR and NONC turned out to be highly correlated with CYTHA (0.81***) and NCYTHA (0.86***), respectively.

**Table 1:** Phenotypic analysis summary of eight yield-related traits from ‘Beauregard’ × ‘Tanzania’ (BT) full-sib family

	NOCR	NONC	TNR	CYTHA	NCYTHA	RYTHA	FYTHA	CI
$\bar{\text{B}}$	2.881	1.934	4.834	12.567	2.208	15.318	15.521	0.472
$\bar{\text{T}}$	0.990	0.763	1.598	4.793	0.869	5.730	41.144	0.107
${\bar{\text{F}}}_{1}$	2.840	1.971	4.795	17.739	2.247	19.980	22.994	0.420
min(F_1_)	1.388	0.513	1.822	6.000	0.572	6.658	13.801	0.140
max(F_1_)	4.494	4.184	7.947	34.226	4.817	37.106	36.880	0.605
$\sigma_{g}^{2}$	0.386	0.277	1.117	27.611	0.313	31.568	23.677	5.88×10^−3^
$\sigma_{ge}^{2}$	0.272	0.213	0.571	17.028	0.310	18.538	34.451	2.30×10^−3^
$\sigma^{2}$	0.686	0.559	1.462	32.271	1.082	35.098	50.836	5.23×10^−3^
*H*^2^ (%)	80.07	78.31	84.59	84.08	70.39	84.73	71.35	88.42

Parental ($\bar{\text{B}}$ and $\bar{\text{T}}$) and progeny $\left({\bar{\text{F}}}_{1}\right)$ means, minimum, and maximum F_1_ means, and genetic $\left(\sigma_{g}^{2}\right)$, genotype-by-environment interaction $\left(\sigma_{ge}^{2}\right)$ and residual $\left(\sigma^{2}\right)$ variance components and heritability (*H*^2^) estimates are shown for eight traits: number of commercial (NOCR), noncommercial (NONC) and total (TNR) roots per plant, commercial (CYTHA), noncommercial (NCYTHA) and total (RYTHA) root yield in t ha^−1^, foliage yield (FYTHA) in t ha^−1^, and commercial index (CI)

### Mapping QTL in the BT population

#### Simulations:

**Figure 2:**
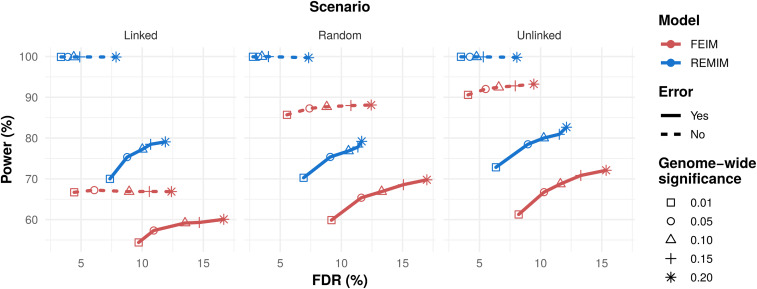
Detection power (in percentage) *vs.* empirical false discovery rate (FDR, in percentage) from QTL mapping analyses of simulated traits in ‘Beauregard’ × ‘Tanzania’ (BT) full-sib family. Each trait was simulated with three QTL (q={1,2,3}) with different heritabilities $\left(h_{q}^{2}=\left\{0.3,0.2,0.1\right\}\right)$ positioned along the BT linkage map (*n* = 298). At least two out of three QTL were linked or not depending on three scenarios (linked, random, and unlinked), with 1000 simulations each scenario. Fixed-effect interval mapping (FEIM, red) and random-effect multiple interval mapping (REMIM, blue) were carried out with (solid lines) and without (dotted lines) the simulated error. FEIM and REMIM used different genome-wide significance thresholds (α={0.20,0.15,0.10,0.05,0.01}, symbols) based on permutation tests or resampling method, respectively. For a ∼95% support interval coverage, power was computed as the proportion of true QTL over the total number of simulated QTL, and FDR as the proportion of false QTL over the total number of mapped QTL.

The BT linkage map based on 298 F_1_ progenies was used to simulate 1000 quantitative traits with three QTL each. We ran FEIM (Equation 1) and REMIM (Equation 4) for each simulated trait in order to assess their power and empirical FDR in three increasingly difficult scenarios. We notice that the proportions of mapped QTL paired to the simulated positions with the highest heritability $\left(h_{1}^{2}=0.3\right)$ were similar regardless of the method and criterion. However, higher proportions of simulated QTL with low heritability $\left(h_{3}^{2}=0.1\right)$ were consistently mapped under the multiple-QTL mapping approach (see Supplemental Material, Table S1). In general, the average absolute difference between the simulated and mapped QTL peak location did not differ whatsoever when comparing models or thresholds. For REMIM, different genome-wide *α* level forward thresholds did not impact power or FDR (results not shown), but varying *α* level backward thresholds was critical. From testing different *d* values for *LOD*−*d* and *LOP*−*d*, we learned that *d* = 1.5 was a good approximation of 95% support interval for both FEIM and REMIM (see Table S2). Based on results for such a support interval, Figure 2 compares different threshold criteria for declaring a QTL during FEIM and REMIM (for forward *α*=0.20 threshold). On the one hand, both FEIM and REMIM have shown a relative control of FDR, with <15% of false discoveries for most *α* levels, regardless of scenario. On the other hand, power differed in up to ∼18% when comparing different *α* levels that delivered <10% FDR. Such a drop in detection power is more noticeable when FEIM is dealing with linked QTL, whereas power barely changes for REMIM across scenarios. Interestingly, even with the most stringent criteria of *α*=0.01 backward threshold for REMIM, we were able to map as many QTL as using *α*=0.20 for FEIM, but with better FDR control (∼7% for REMIM in comparison to >15% for FEIM). By fitting the model without the simulated residual error in the “unlinked” scenario, we observed ∼92% power for FEIM. The small difference in comparison to ∼100% power for REMIM is likely due to the fact that the QTL genotype effects were simulated based on the REMIM additive relationship model (Equation 4). Despite the relative bias, FEIM failure in separating linked QTL became more evident in the “random” and “linked” scenarios. As a consequence, detection power plateaus ∼87% and 66% were observed, while REMIM exhibited ∼100% power (Figure 2).

#### Yield-related traits:

**Figure 3:**
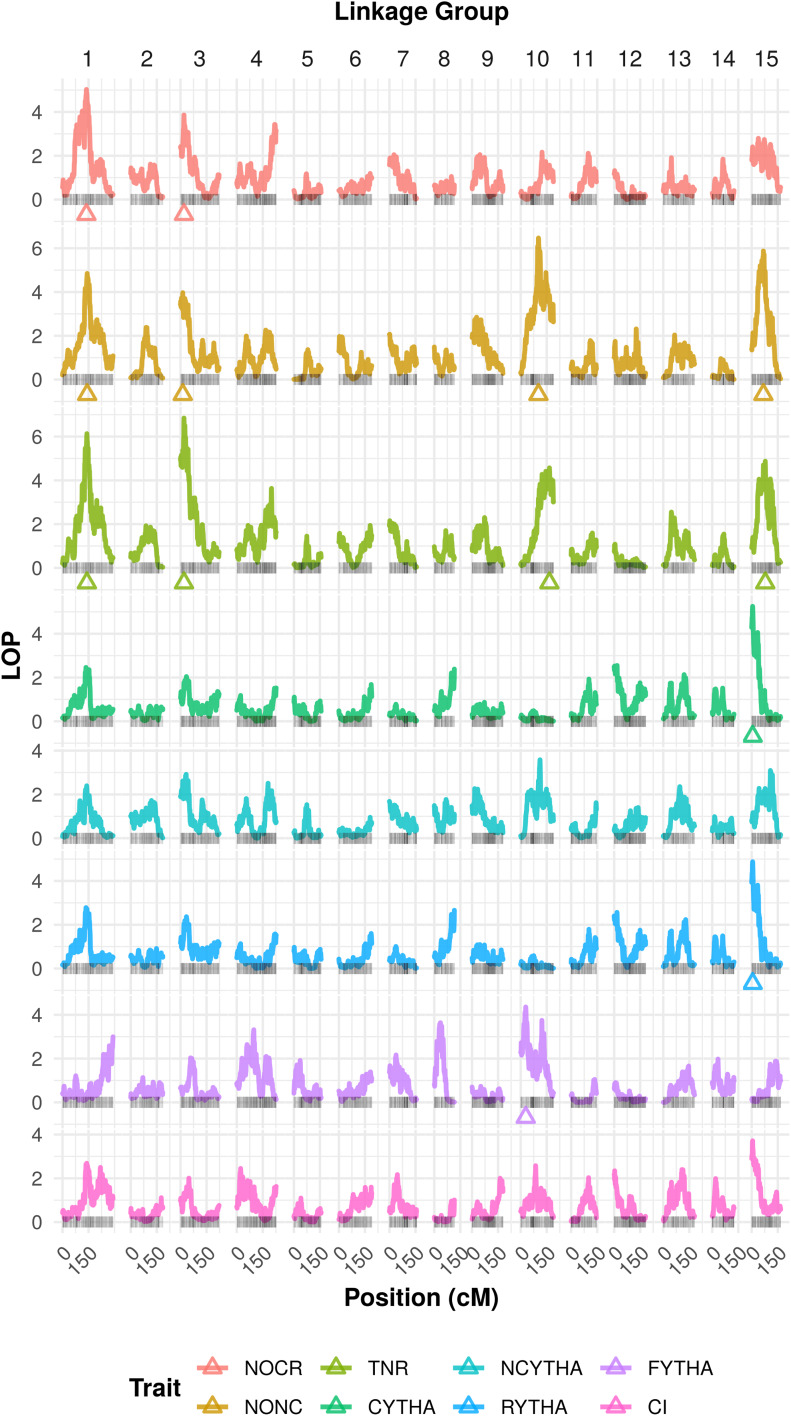
Logarithm of *P*-value (*LOP*) profiles from random-effect multiple interval mapping (REMIM) of eight yield-related traits from ‘Beauregard’ × ‘Tanzania’ (BT) full-sib family. Triangles show the QTL peak location. Trait abbreviations: number of commercial (NOCR), noncommercial (NONC), and total (TNR) roots per plant, commercial (CYTHA), noncommercial (NCYTHA) and total (RYTHA) root yield in t ha^−1^, foliage yield (FYTHA) in t ha^−1^, and commercial index (CI).

We adopted genome-wide *α* levels of 0.20 and 0.05 as the respective forward and backward thresholds for detecting QTL in eight yield-related traits in the BT population using REMIM (Equation 4), such that respective resampling-based *P*-value thresholds were defined as 5.83×10^−4^ and 1.42×10^−4^. In total, 13 QTL were identified (Figure 3), with *P*-values ranging from 1.42×10^−7^ (QTL 2 for TNR) to 1.37×10^−4^ (QTL 2 for NOCR) (Table 2). The number of QTL per trait ranged from one (CYTHA, RYTHA and FYTHA) to four (NONC and TNR); NOCR had two QTL, and no QTL were found for NCYTHA and CI. Four LG harbored QTL regions: LGs 1, 3, and 10 harbored three QTL each, and LG 15 harbored four QTL. Approximate 95% support intervals computed as *LOP*−1.5 (see Figure S1) showed that QTL were colocalized mostly within each LG. QTL peaks for NOCR, NONC, and TNR can be found from 137.60 to 142.07 cM on LG 1, and from 13.11 to 20.18 on LG 3. On LG 15, QTL peaks were localized either at 67.20 and 78.04 cM for NONC and TNR, respectively, or at 5.34 cM for both CYTHA and RYTHA. QTL variance $\left(\sigma_{q}^{2}\right)$ and heritability $\left(h_{q}^{2}\right)$ estimates from Equation 3 are shown in Table 2, where the subscript *q* denotes the QTL number for a specific trait. QTL heritabilities ranged from 8.99 (QTL 3 for TNR) to 22.04% (QTL 2 for TNR), representing the proportion of the total variance explained by that QTL, conditional to all the other QTL in the model. Out of 13 QTL, 4 were considered major QTL $\left(h_{q}^{2}>15\text{\%}\right)$, which happened as pairs of colocalized QTL at the beginning of LGs 3 for NOCR/TNR and 15 for RYTHA/CYTHA (see Figure S2). Altogether, multiple QTL explained as much as 35.63%, 49.19%, and 55.06% of the total variance for NOCR, NONC, and TNR, respectively. In order to compare these QTL detection results with FEIM, we adopted an *α*=0.05, so that permutation-based LOD score thresholds ranged from 7.63 to 7.85, depending on the trait (see Figure S4). A total of 12 QTL were mapped (see Table S3): one for each CYTHA, RYTHA, and FYTHA; two for NOCR; three for TNR; and four for NONC. No QTL were found for NCYTHA and CI either. The same four LGs harbored QTL: LGs 1 and 10 had two QTL each, LG 3 had three and LG 15 had five QTL, with the most significant QTL (adjusted *R*^2^ > 11) found on LGs 1, 3, and 15. In comparison with REMIM, FEIM did not detect QTL for NONC or TNR on LGs 1 and 10, respectively. Instead, it allowed two QTL on LG 15 for NONC (one at 56.86 cM and another at 119.08 cM).

**Table 2:** Random-effect multiple interval mapping (REMIM) of yield-related traits from ‘Beauregard’×‘Tanzania’ (BT) full-sib family

Trait	QTL	LG	Position (cM)	Score	*P*-value	$\sigma_{q}^{2}$	$h_{q}^{2}$ (%)
NOCR	1	1	137.60 (99.43–152.87)	222.89	9.35 × 10^−6^	0.0622	13.70
	2	3	20.18 (0.00–49.27)	172.52	1.37 × 10^−4^	0.0996	21.93
NONC	1	1	142.07 (128.08–159.30)	207.83	1.42 × 10^−5^	0.0447	10.11
	2	3	13.11 (0.00–51.33)	165.75	1.07 × 10^−4^	0.0420	9.50
	3	10	102.26 (96.50–113.55)	267.28	3.40 × 10^−7^	0.0647	14.63
	4	15	67.20 (39.10–78.04)	247.92	1.33 × 10^−6^	0.0661	14.95
TNR	1	1	140.43 (128.08–152.87)	251.13	7.36 × 10^−7^	0.1789	10.97
	2	3	20.18 (13.11–43.69)	279.18	1.42 × 10^−7^	0.3595	22.04
	3	10	165.43 (102.26–187.27)	192.09	2.69 × 10^−5^	0.1467	8.99
	4	15	78.04 (35.50–119.08)	207.07	1.34 × 10^−7^	0.2131	13.06
CYTHA	1	15	5.34 (0.00–34.27)	242.24	5.62 × 10^−6^	6.3128	19.93
RYTHA	1	15	5.34 (0.00–35.50)	226.56	1.32 × 10^−5^	6.9177	18.97
FYTHA	1	10	29.09 (16.12–134.37)	203.16	4.4 × 10^−5^	2.1077	14.78

Linkage group (LG), map position (in centiMorgans) and its ∼95% support interval (within parenthesis), score statistic and its corresponding *P*-value, variance $\left(\sigma_{q}^{2}\right),$ and heritability ($h_{q}^{2}$, in percentage) of mapped QTL using resampling-based genome-wide significance *P*-value threshold of 0.05 (backward elimination)

Trait abbreviations: number of commercial (NOCR), noncommercial (NONC) and total (TNR) roots per plant, commercial (CYTHA), noncommercial (NCYTHA) and total (RYTHA) root yield in t ha^−1^, foliage yield (FYTHA) in t ha^−1^, and commercial index (CI).

**Figure 4:**
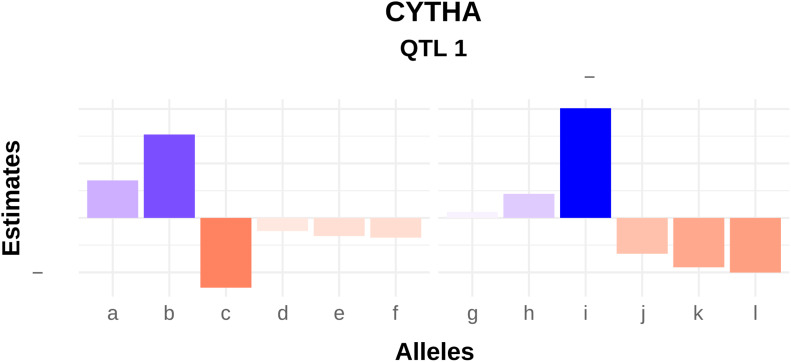
Additive allele effects from the decomposed best linear unbiased predictions (BLUPs) for the QTL 1 (on linkage group 15 at 5.27 cM) of commercial root yield in t ha^−1^ (CYTHA) in a hexaploid sweetpotato full-sib family (‘Beauregard’ × ‘Tanzania’). Letters represent each of the six haplotypes from each parent.

From REMIM, additive allele effects (see Table S4) were derived from the QTL genotype BLUPs (Equation 3). In general, although both parents have shown allele contributing to either decreasing or increasing the trait means, ‘Beauregard’ seemed to contribute more, increasing the number of roots, while ‘Tanzania’ exhibited major alleles increasing root yield. These effects represent the parental contributions to the population mean, *i.e.*, how much one adds to, or subtracts from, the mean, given 1 of the 400 possible genotypes. For instance, Figure 4 shows the additive allele effects of QTL 1 for CYTHA. Inferences on which alleles contribute more to the mean as well as which ought to be selected for breeding purposes are straightforward. For example, individuals with the haplotypes *b* from ‘Beauregard’ and *i* from ‘Tanzania’, and without the haplotypes *c* and *j* through *l* from the respective parents will have the highest QTL-based breeding value estimates for CYTHA. By computing QTL-based breeding values, we could hypothesize on the genetic basis of trait correlation (see Figure S3). For instance, NOCR and NONC were highly correlated (0.51***) given a couple of colocalized QTL. While TNR is significantly correlated to NOCR/NONC (>0.76***), smaller correlation was observed between TNR and CYTHA/RYTHA (<0.15*). Interestingly, QTL-based breeding values for FYTHA did not seem to correlate to any other root-related trait. Finally, the absolute positive correlation of 1.00*** between predicted means from CYTHA and RYTHA (Figure 1) could be explained by a single colocalized QTL, since the correlation between QTL-based breeding values was also very high (0.99***).

### Candidate genes underlying major QTL

We elected to examine putative candidate genes under two QTL with the highest heritabilities: the QTL for TNR on LG 3 (colocalized for NOCR and NONC) and the QTL for CYTHA on LG 15 (colocalized for RYTHA). The QTL peak on LG 3 was positioned at 1,591,872 bp relative to *I. trifida* genome (see Table S5), and 75 genes were found within a ∼500-kb window around this peak. Examination of functional annotation of these genes, coupled with expression profiles in leaves, as well as a time course of developing roots in both ‘Beauregard’ and ‘Tanzania’ (Gemenet *et al.* 2020) (see File S3, File S4), revealed three candidate genes of interest (see Figure S5). The first *I. trifida* gene, *itf03g02930*, encodes a homolog of SKU5, a glycosyl phosphatidylinositol modified protein in *Arabidopsis thaliana*; the second candidate gene, *itf03g03280*, encodes a protein with sequence similarity to annexin (ANN1 and ANN2); and the last candidate gene, *itf03g03460*, encodes a protein similar to the WUSCHEL homeobox family protein (AtWOX13). In general, these genes were expressed at a low level in leaves, but highly in roots. Six additional genes were found to be differentially expressed in ‘Beauregard’ storage roots relative to fibrous roots across the time course, while only a single gene was differentially expressed in ‘Tanzania’ (see File S5).

On LG 15, a major QTL for CYTHA, with the peak at 477,772 bp, spanned positions from 21,822 to 1,915,814 bp (see Table S5) and over 300 genes. As this was too large a distance to manually curate candidate genes responsible for the trait, we restricted our manual review to 25 genes distal and proximal to the most significant marker. Within this region, two genes encoded functions that may be associated with storage root development and had expression profiles that supported a role in storage root development (see Figure S5, File S3, File S4). The hormone ethylene has diverse roles in cell proliferation and elongation, and the *itf15g01020* gene encodes a protein with similarity to the *A. thaliana CONSTITUTIVE TRIPLE RESPONSE 1* gene (*CTR1*) with functions in the ethylene signaling pathway. This gene was expressed in leaves but expressed at twice the levels in developing roots. Storage roots are grown for their high starch content, and *itf15g01120* encodes a protein with similarity to starch branching enzyme 2.2. This gene was expressed in leaves and roots with the highest expression levels detected in storage, not fibrous nor developing roots. Analysis of differentially expressed genes in storage *vs.* fibrous roots of ‘Beauregard’ and ‘Tanzania’ within this QTL region revealed 39 unique differentially expressed genes in ‘Beauregard’ across the time course, and 12 unique differentially expressed genes in ‘Tanzania’ (see File S5).

## Discussion

### Polyploid single- *vs.* multiple-QTL models

QTL mapping in autopolyploid species has been limited to a fixed-effect interval mapping (FEIM) model proposed for tetraploids (Hackett *et al.* 2001, 2014) and also expanded for hexaploids (van Geest *et al.* 2017). Consisting of a single-QTL model, 2*m*−2 main effects are fitted (*m* is the ploidy level), and this model is compared to a null model (with no QTL) using LRT, ultimately expressed as LOD scores. Permutation-based genome-wide significance LOD thresholds are then used to declare a QTL. Trying to fit additional QTL into FEIM model could rapidly lead it to over-parameterization, since each QTL requires as many as 6 (for tetraploids), 10 (for hexaploids), or 14 (for octoploids) main effects to be tested and estimated in such a fixed-effect multiple-QTL model. Furthermore, new rounds of permutation tests, based on a model with QTL, would need to be carried out in order to provide an updated LOD score threshold (Klaassen *et al.* 2019). In contrast, the random-effect multiple interval mapping (REMIM) model presented here is designed to fit multiple random-effect QTL by estimating only one single parameter $\left(\sigma_{q}^{2}\right)$ per QTL. Score statistic tests are performed in order to assess whether a QTL variance component is zero or not, conditional to other QTL in the model. These tests provide an approach for comparing two nested models with the reduced model having a random effect excluded, similar to what restricted LRT (RLRT) would do. However, (R)LRT is more prone to numerical errors because the null hypothesis $\left(H_{0}:\sigma_{q}^{2}=0\right)$ falls on the boundary of the parameter space, whereas score-based methods can be robust to eventual misspecification of the distribution of random effects (Verbeke and Molenberghs 2003). A score-based resampling method (Zou *et al.* 2004) was used for setting genome-wide significance thresholds, which facilitates a forward–backward search to identify an optimal multiple QTL-model in a computationally tractable manner.

Here, we used the BT population genetic map to simulate quantitative traits based on multiple QTL with different heritabilities each, in order to compare FEIM and REMIM performances under three increasingly difficult scenarios (Figure 2). Both approaches would potentially detect similar number of QTL in case they were all unlinked. However, despite the small bias created by the way QTL were simulated (based on REMIM model), FEIM showed a relative loss of power. Multiple-QTL model approaches have proven to provide greater power and better FDR control than single-QTL models for both univariate (Zeng *et al.* 1999; Laurie *et al.* 2014) and multivariate (Da Costa E Silva *et al.* 2012b) models, due mostly to the differences in detecting QTL with smaller effects. In fact, this is rather expected as a multiple-QTL model has a smaller residual variance, which helps to detect additional QTL. Multiple-QTL models are also supposed to improve detection of more than one QTL on the same LG (Mayer 2005), as they are usually hard to separate from each other due to the large extension of linkage disequilibrium in mapping populations. For polyploids, a nonoptimized approach of using residuals from a fitted single-QTL model as phenotypic data to find a second linked QTL has been proposed (Mengist *et al.* 2018), as it requires additional manual steps. In contrast, the forward–backward search employed here has been shown to be optimized to detect linked QTL. The consistently superior results in comparison to FEIM pointed out that the linear score statistics behaved well as part of our algorithm, and the impact of using different *α* level thresholds for QTL detection was also assessed here. In QTL mapping analysis, it is important to have a reasonable balance between detection power and FDR, as we are interested in mapping as many true QTL as possible. When deciding on which *α* level to adopt, one should consider the goals of the study, *i.e.*, whether it is intended to use a few very reliable QTL for marker-assisted breeding, or to discover as many QTL-related putative genes as possible for further validation. Although one could use a more relaxed criteria in order to increase the power of detection while still maintaining an acceptable level of FDR, REMIM with respective forward and backward *α* levels of 0.20 and 0.05 seemed reasonable.

### QTL mapping for yield traits in sweetpotato

Most of the linkage and QTL mapping work done for sweetpotato so far has relied on strategies based on a double pseudotestcross approach for diploid species (Grattapaglia and Sederoff 1994). For example, separate parental maps have been built based on this diploid-based simplification, using qualitative marker systems such as randomly amplified polymorphic DNA (RAPD; Ukoskit and Thompson 1997), amplified fragment length polymorphism (AFLP; Kriegner *et al.* 2003; Cervantes-Flores *et al.* 2008a; Nakayama *et al.* 2012), retrotransposon insertion polymorphisms (Monden *et al.* 2015), and simple sequence repeats (SSR; Kim *et al.* 2017). A recent map was developed from a selfing population and used only single-dose SNPs, resulting in higher marker saturation in comparison to the previous maps (Shirasawa *et al.* 2017), though the map was still not integrated. In some of these cases, QTL mapping analyses were performed for several traits, related mostly to quality (Cervantes-Flores *et al.* 2011; Zhao *et al.* 2013; Yu *et al.* 2014; Kim *et al.* 2017) and resistance to biotic stresses (Cervantes-Flores *et al.* 2008b; Yada *et al.* 2017a). For yield-related traits, only two studies have been reported to date (Chang *et al.* 2009; Li *et al.* 2014). The use of DNA markers with unknown DNA sequence limited our ability to compare their results with *I. trifida* and *I. triloba* genomes (Wu *et al.* 2018), and, ultimately, with our present QTL study (see Table S5). Moreover, although these diploid-based strategies were the state-of-the-art at that time for qualitative marker-based, low density genetic maps, they imposed significant restrictions on statistical power for QTL detection and its genetic interpretation.

Recently, more improved methods and computational tools that take into account autopolyploid complexity for dosage SNP calling (Voorrips *et al.* 2011; Serang *et al.* 2012; Schmitz Carley *et al.* 2017; Gerard *et al.* 2018) and integrated linkage map construction (Hackett *et al.* 2016; Bourke *et al.* 2018; Mollinari and Garcia 2019) have become available, mostly dedicated to tetraploids. Taking advantage of the newly developed MAPpoly package, Mollinari *et al.* (2020) built the first integrated genetic map for sweetpotato, from the BT population used here. For a hexaploid species, this has opened up new opportunities for more interpretable QTL genetic models due to MAPpoly implementation of a HMM that delivers QTL genotype conditional probabilities along a fully integrated genetic map (Mollinari and Garcia 2019). Based on this map, we detected 13 QTL (Figure 3) using REMIM, with QTL heritabilities ranging from 8.99 to 22.05% (Table 2, see Figure S2). Most of these QTL were also mapped among the 12 QTL using FEIM (see Figure S4), with proportion of variance explained (PVE) ranging from 8.42 to 12.43% (see Table S3). Based on the double pseudotestcross approach, previous studies found nine QTL for storage root yield (17.7%≤PVE≤59.3%) (Li *et al.* 2014), seven QTL for root and top (foliage) weight (16.0%≤PVE≤29.5%), plus one QTL for root number (PVE=14.8%) (Chang *et al.* 2009). Because of likely estimation bias due to reduced population sizes (*n* < 200), and the use of not very informative markers and linkage maps, these previous PVE findings are hard to compare with our results.

Although number of roots seemed to be as heritable as root yield (Table 1), only one colocalized QTL was detected for CYTHA/RYTHA. These traits are likely more complex in terms of their genetic architecture, though. That is, not only number of roots contributes to yield, but also size and composition, so we can expect that more regions are involved in root yield, in addition to those involved in number of roots. Nevertheless, Yada *et al.* (2017b) found a rather low trait heritability (likely individual-basis) for commercial root yield (*H*^2^ = 24%) among 278 full-sibs of a cross between ‘New Kawogo’, a Ugandan landrace, and ‘Beauregard’, possibly due to stronger G × E interaction, which adds to the trait complexity. Here, G × E interaction seemed important for all traits, and its consequences to QTL mapping and breeding will be explored in future studies. As QTL mapping targets major QTL, usually stable across environments, most of the minor ones must have gone undetected. Moreover, additional genetic variation could be due to higher-order allele interactions and genetic epistasis, which the current models do not account for. Colocalized QTL among number of roots and yield traits explain some of the correlations among QTL-based breeding values (see Figure S3), partially explaining the correlations among the predicted means for these traits (Figure 1). Based on the correlation between QTL-based breeding values, FYTHA does not seem to be useful in indirect selection for CYTHA, as suggested previously (Chang *et al.* 2009).

‘Beauregard’ and ‘Tanzania’ contributed more importantly with positive and negative major effects, respectively. However, the presence of both favorable and unfavorable QTL alleles were observed in either parents (see Table S4), which possibly explains the presence of transgressive segregants for all traits. Transgression in polyploids seems to be due to cumulative complementary alleles not only at different loci (Tanksley 1993), but also from the same QTL. In fact, increased heterozygosity has been suggested as one of the major forces of polyploid evolutionary success, as a broader allele repertoire may result in the variation of gene expression and regulation needed to thrive in more diverse environmental conditions (Van de Peer *et al.* 2009). As an example, ‘Tanzania’ exhibited alleles contributing to increase CYTHA from a major QTL (Figure 4), although this landrace was not very productive in our environments overall. The additive effects are the most important from a breeding point-of-view, and their estimation provides straightforward direction on which alleles to select. Simpler biallelic-based models proposed previously (Hackett *et al.* 2014; Chen *et al.* 2018) may be used to estimate other interactions. The effective use of these allele interactions in QTL detection and breeding remains limited, though. As noted by Gallais (2003), estimating multi-allelic interactions reliably would require larger populations.

Several studies have looked at genes involved in storage root initiation and development in sweetpotato as reviewed by Khan *et al.* (2016). The storage roots differentiate from lateral roots by development of cambia around the protoxylem and secondary xylem, while lignification of the steles of some lateral roots inhibits this transformation (Villordon *et al.* 2012). Using the expression profile of the parents of the current mapping population, we found genes in leaves and roots (see Figure S5) related to root directional growth (*e.g.*, SKU5, *itf03g02930* homolog; Sedbrook *et al.* 2002) and lateral development (*e.g.*, AtWOX13, *itf03g03460* homolog; Deveaux *et al.* 2008) as well as with sugar transport to the root tip (*e.g.*, ANN1 and ANN2, *itf03g03280* homolog; Wang *et al.* 2018) within the QTL region on LG 3 associated with number of storage roots. Thus, both root restructuring and carbon supply is likely involved in the number of lateral root that transform to storage root. Other genes such as MADS-box (*e.g.*, *itf03g02230* homolog; Kim 2002), expansin (EXP, *itf03g05010* homolog), and BEL1-like homeodomain (*e.g.*, *itf03g02670* homolog; Ponniah *et al.* 2017) have been strongly implicated in storage root formation and development in sweetpotato, and were found within the QTL region on LG 3. On the QTL related to storage root weight on LG 15, we found genes related with the hormonal control of cell proliferation (*e.g.*, *CTR1*, *itf15g01020* homolog; Street *et al.* 2015) and with starch biosynthesis (*e.g.*, starch branching enzyme 2.2, *itf15g01120* homolog; Li *et al.* 2014). The association between these and other differentially expressed genes listed in this study (see File S5) is yet to be defined, and suggests the complex nature of storage root formation and development.

### Final considerations

Here, we present a stepwise-based algorithm for multiple-QTL model selection in full-sib populations of autopolyploid species with a fully integrated map, from which QTL genotype conditional probabilities can be calculated. The use of score statistics is a key component of this new method, which depends on a dynamic and fast-computing test for model selection during the QTL detection process. Simulations were performed in order to assess the impact of using different threshold criteria and to provide some empirical sense on how to use the method in practice. REMIM has been carried out in a hexaploid sweetpotato population to detect major loci contributing to the variation of yield-related traits that may be targeted in molecular-assisted breeding. The use of random-effect models has created the context for fitting multiple QTL, providing straightforward information on variance components, important for computing QTL heritabilities. Finally, QTL genotype predictions (BLUPs) allowed us to estimate additive effects for characterizing major allele contributions, and compute QTL-based breeding values that can be used for performing selection. This novel approach may enable more complex models, such as those accounting for interaction among QTL as well as multiple traits or multiple environments in order to study shared genetic control in different traits/environments and G × E interaction at QTL level. Such a model can also be expanded in order to consider multi-parental designs and double reduction or preferential pairing as long as the reconstructed haplotypes can be used to inform on shared alleles IBD. For these more complex models, one could expect additional computational cost, for which further investigation is needed. Understanding the genetic architecture of root yield and other traits related to quality and resistance to biotic and abiotic stresses represents great opportunity for improving characteristics of interest in sweetpotato and other polyploids. Most of these important traits are polygenic in nature and only assessed later in a breeding program, where marker-assisted selection could help to speed up the process.
